# Hydrothermal Preparation and Characterization of Ultralong Strontium-Substituted Hydroxyapatite Whiskers Using Acetamide as Homogeneous Precipitation Reagent

**DOI:** 10.1155/2014/863137

**Published:** 2014-01-28

**Authors:** Jianqiang Xu, Yaoqi Yang, Rong Wan, Yuhui Shen, Weibin Zhang

**Affiliations:** Department of Orthopaedics, Shanghai Institute of Orthopaedics and Traumatology, Shanghai Ruijin Hospital, Shanghai Jiaotong University School of Medicine, 197 Ruijin Er Road, Shanghai 200025, China

## Abstract

The ultralong strontium- (Sr-) substituted hydroxyapatite (SrHAp) whiskers were successfully prepared using acetamide as homogeneous precipitation reagent. The effect of the Sr substitution amount on the lattice constants and proliferation of human osteoblast cells (MG-63) was further investigated. The results showed that the SrHAp whiskers with diameter of 0.2–12 *μ*m and ultralong length up to 200 *μ*m were obtained and the Sr substitution level could be facilely tailored by regulating the initial molar ratio of Sr/(Sr + Ca) in raw materials. The Sr^2+^ replaced part of Ca^2+^ and the lattice constants increased apparently with the increase of the Sr substitution amount. Compared with the pure HAp whiskers, the Sr substitution apparently stimulated the proliferation of MG-63 at certain extracted concentrations. Our study suggested that the obtained SrHAp whiskers might be used as bioactive and mechanical reinforcement materials for hard tissue regeneration applications.

## 1. Introduction

The hydroxyapatite [Ca_10_(PO_4_)_6_(OH)_2_, HAp] bioceramics are common bone graft materials and are widely used in biomedical fields due to their excellent biocompatibility, osteoconductive properties, and similarity to the inorganic component of natural bones and teeth [1–3]. However, the mechanical property of HAp materials is unsatisfactory, especially for the toughness, which has severely hindered their wider clinical applications [4, 5]. Moreover, the HAp bioceramics are largely considered to be lacking in the osteoinductive ability, which may impact repair capacity for large bone defects, nonunions, and follow-up function restoration [6]. So far, many strategies have been developed to solve these problems, such as using ZrO_2_ metals and carbon nanotubes. as mechanical reinforcement [5, 7, 8]. However, these kinds of reinforcements are bioinert and/or nonbiocompatible, which might reduce the bioactivity and biocompatibility of the implants. It is realized that the HAp whiskers might possess great prospect to be used as the mechanical reinforcements because of their excellent biocompatibility [4, 9–15]. It is considered that the traditional HAp whiskers are also lacking in the ability to stimulate the formation of new bone. It is well known that, as a trace element in human body, the strontium (Sr) plays distinctly dual roles in stimulating bone formation and inhibiting bone resorption [2, 16, 17]. The mechanism is thought to lie in Sr^2+^ ions, which not only can apparently promote osteoblast-related gene expression and the alkaline phosphatase (ALP) activity of mesenchymal stem cells (MSCs) but also can decrease the differentiation of osteoclasts [18]. As a newly developed drug to prevent osteoporosis, the Sr ranelate has been confirmed to possess dual effects of stimulating osteoblast differentiation and inhibiting osteoclast activity and bone resorption, and ultimately reduce the incidence of fractures in osteoporotic patients [18, 19]. Moreover, the partial substitution of Ca by Sr can apparently improve the biological properties of phosphate and silicate bioceramics and bioglasses [16, 17, 20]. Therefore, compared with the traditional HAp whiskers, the Sr-substituted HAp whiskers (SrHAp) might possess excellent mechanical and biological properties.

The previous study suggested that the hydrolysis rate of acetamide was apparently lower than that of the traditional urea additive in the hydrothermal homogeneous precipitation method, which might be of great benefit to the rapid growth of whiskers at a low supersaturation [14, 15, 21]. In this study, the ultralong SrHAp whiskers were hydrothermally prepared using acetamide as homogeneous precipitation reagent. Then the effect of Sr substitution on morphologies, phases, lattice constants, and osteoblast proliferation of the products was further studied.

## 2. Materials and Methods

### 2.1. Synthesis and Characterization of SrHAp Whiskers

The SrHAp whiskers designed Sr/(Ca + Sr) molar ratios of 0.025, 0.05, and 0.1 were hydrothermally synthesized using acetamide as homogeneous precipitation reagent. Aqueous solutions containing 50 mmol (Ca^2+^ + Sr^2+^) ions and 29.94 mmol HPO_4_^2−^ were prepared by dissolving analytical grade reagents of Ca(NO_3_)_2_·4H_2_O, Sr(NO_3_)_2_, and NH_4_H_2_PO_4_ in distilled water with 1 mol/L acetamide. The 0.1 mol/L HNO_3_ solution was used to adjust the pH to around 2.75 to obtain clear solutions. Then 85 mL of the obtained solution was transferred into 100 mL Teflon autoclaves and heated at 180°C for 10 h, followed by cooling to room temperature naturally. After hydrothermal reaction, the obtained suspensions were filtrated and washed with distilled water and anhydrous ethanol for 3 times, respectively, and then dried at 120°C for 24 h. The pure HAp whiskers in the absence of Sr substitution were prepared as the control sample via the similar method.

The obtained products were characterized by X-ray diffraction (XRD: D/max 2550 V, Rigaku, Japan) with mono-chromated Cu-K*α* radiation. The lattice constants were calculated from the well-determined positions of the intense XRD diffractions that were processed by MDI Jade 6.1 software (Materials Data Inc., USA) [22]. The whiskers were also characterized using the Fourier transform infrared spectroscopy (FTIR: Nicolet Co., USA). The morphology and size of the whiskers were observed on field emission scanning electron microscopy (FESEM: JSM-6700F, JEOL, Japan), and the chemical compositions of the powders were analyzed by inductively coupled plasma atomic emission spectroscopy (ICP-AES; VISTA AX, Varian Co., USA) after dissolving the whiskers in 0.1 mol/L hydrochloric acid aqueous solution.

### 2.2. Effect of Ionic Products from SrHAp Whiskers on MG-63 Proliferation

The ionic extract method is a widely used international standard to evaluate the effect of the chemical compositions on cell biological responses, which can effectively avoid the extra effects that came from the material morphologies via directly incubating the materials with cells [2, 23]. Herein, the human osteoblast cells (MG-63, Cell bank, Shanghai, China) were cultured in the medium consisting of a-MEM (89%, GIBCO, Invitrogen, Grand Island, NY, USA), fetal bovine serum (10%, FBS; Gibco, USA), and penicillin streptomycin (1%, PS; Gibco, USA). To prepare the extracts, a stock solution of 50 mg/mL was first prepared by adding the whiskers into DMEM (GIBCO Invitrogen, Grand Island, NY) culture medium. After incubation at 37°C for 24 h, the mixtures were centrifuged and the supernatants were collected. The serial diluted extracts (25 and 12.5 mg/mL) were prepared by diluting the stock solutions with serum-free DMEM. Subsequently, these extracts were sterilized by filtration through 0.2 *μ*m filter membranes for cell culture experiments. The ion concentrations of the extracts were measured by ICP-AES.

The MG-63 was seeded in 96-well plates at a density of 5 × 10^3^ cell/well and cultured by incubation at 37°C for 5 days with 5% CO_2_ and 95% air at 100% RH. The medium in the well was then replaced by the prepared extracts. The culture medium was changed every 2 days. After 5 days of culture, 10 *μ*L (5 mg/mL) of 3-(4,5-dimethylthiazol-2-yl)-2,5-diphenyl tetrazolium bromide (MTT, Dojindo, Kumamoto, Japan) plus 100 *μ*L of DMEM were added into each well. After additional incubation for 4 h, the MTT solution was removed and replaced with 100 *μ*L of dimethylsulfoxide (DMSO). After 10 min of slow shaking (Vibramax 100, Metrohm, USA), the absorbance was read at 570 nm against the reference value at 630 nm, and the results were expressed as optical density (OD). All experiments were done in triplicate to obtain the average data.

### 2.3. Statistical Analysis

Data were analyzed for statistical significance using an analysis of variance. Differences at *P* values of <0.05 were considered significant.

## 3. Results and Discussion

### 3.1. Characterization of SrHAp Whiskers

**Figure 1:**
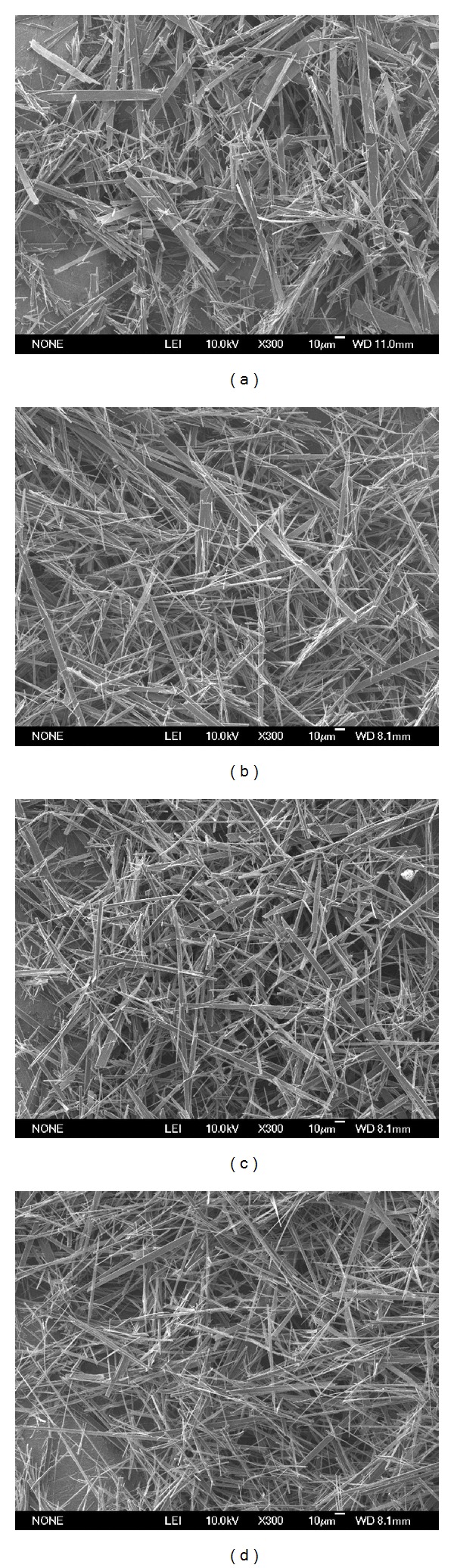
SEM images of the obtained HAp (a), Sr2.5HAp (b), Sr5HAp (c), and Sr10HAp (d) whiskers; Bar = 30 *μ*m.

Figure 1 shows the morphologies of the obtained products. It is clear to see that all of the products were whisker-like morphology with diameter of 0.2–12 *μ*m and ultralong length up to 200 *μ*m. Almost none of the particles were observed. The SEM observation suggested that the Sr substitution did not alter the morphologies of the products. In the homogeneous precipitation method, the reagent of urea is usually applied as the additive to raise the pH value to drive the nucleation and growth of HAp crystals under hydrothermal treatment [1, 24]. Compared with the additive of urea, the acetamide possesses a lower hydrolysis rate under the required hydrothermal conditions, which allows better and easier control and gives rise to rapid growth of whiskers at low supersaturation [21]. Moreover, the average of the diameters and lengths of the SrHAp whiskers were almost 1.47–1.85 and 0.46–0.61 times higher than those of the HAp whiskers, respectively. The results suggested that the Sr-substitution might increase the size of the whiskers. However, the mechanism needs to be further investigated in detail.

**Figure 2:**
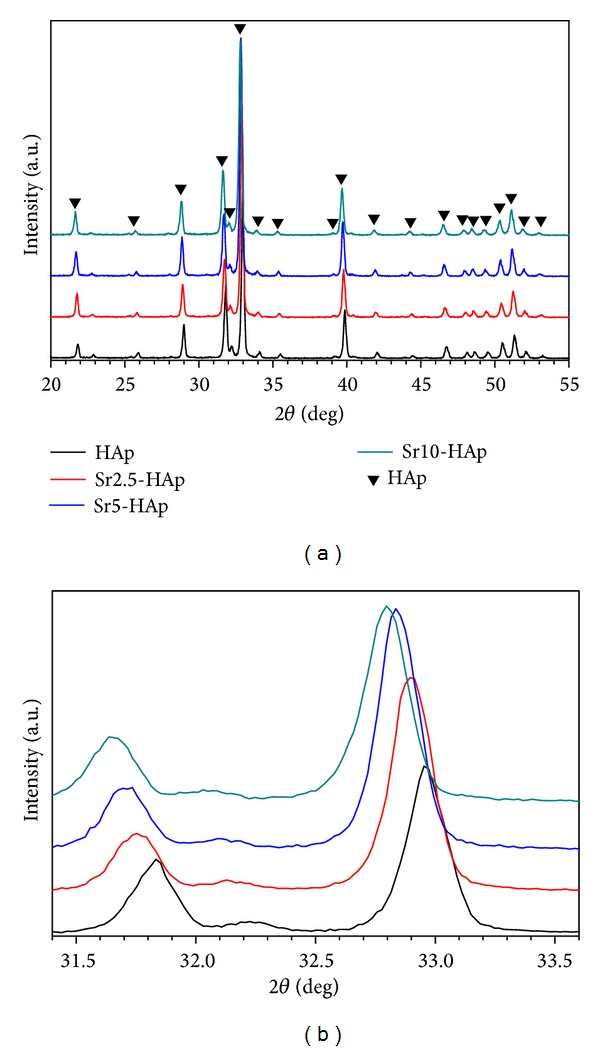
XRD patterns of the obtained HAp and Sr-substituted HAp whiskers.

Figure 2 presents the XRD patterns of the obtained HAp and Sr-substituted HAp whiskers. The results showed that all of the products could be well identified as pure HAp phase (JCPDS card: NO. 09-0432). Compared with the pure HAp whiskers, the small angle XRD scanning results (Figure 2(b)) clearly showed that the corresponding peaks of the obtained Sr-substituted HAp whiskers shifted to lower degree. Moreover, with the increase of the Sr substitution level, the shifting extent increased apparently, suggesting the increase of the lattice constants (Table 1) [2]. The calculated lattice constants based on the XRD determination results validated that the lattice constants of the prepared Sr-substituted HAp whiskers were larger than those of the pure HAp whiskers (Table 1). The increase of the lattice constants was attributed to the replacement of the Ca^2+^ ions by bigger diameter of Sr^2+^ ions [2]. The shifting of the patterns and the deviation of lattice constants suggested that the Sr^2+^ ions replaced and occupied the Ca^2+^ crystal positions of the HAp [2, 3]. However, the peak intensities of the (2 1 1) and (3 0 0) reflections were different from the standard values, which was attributed to the preferential orientation growth of the HAp whiskers.

**Figure 3:**
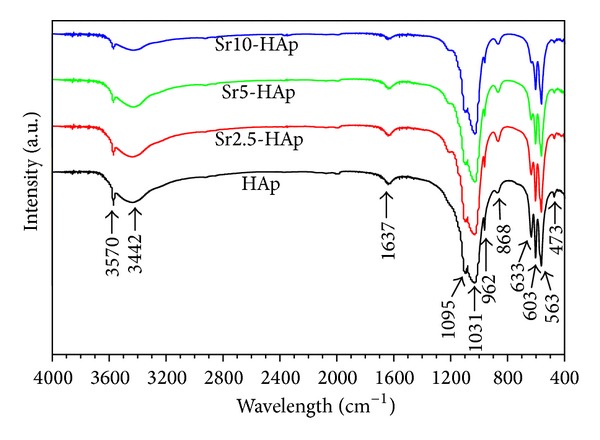
The FTIR spectra of the obtained HAp and Sr-substituted HAp whiskers.

Figure 3 reveals the FTIR spectra of the obtained HAp and Sr-substituted HAp whiskers. The spectra accord well with the reported FTIR data for HAp. The peaks at 473, 563, 603, 962, 1031, and 1095 cm^−1^ were the characteristic bands for PO_4_^3−^ [1–3]. The peaks at 3442 and 1637 cm^−1^ were assigned to the bending mode of the absorbed water. The peak at 868 cm^−1^ was attributed to the carbonate ion in the B-site, which might come from the dissolved carbon dioxide in aqueous solution [16]. The peaks at 3570 and 633 cm^−1^ were the characteristic OH bands of HAp [3]. The FTIR results further confirmed that the positions of the peaks were not affected by Sr substitution and the products were HAp crystals.

The ICP-AES analytic results of the obtained whiskers are presented in Table 1. The results showed that the prepared Sr-HAp whiskers contained the substituted ions of Sr and the substitution amount of Sr in the obtained Sr-HAp whiskers increased obviously with the increase of the initial molar ratio of Sr/(Sr + Ca) in raw materials. In addition, the (Ca + Sr)/P molar ratio of the obtained Sr-substituted HAp whiskers was between 1.61 and 1.65, which was slightly deviated from the stoichiometric HAp (Ca/P = 1.67).

### 3.2. Effect of Ionic Products from SrHAp Whiskers on MG-63 Proliferation

**Figure 4:**
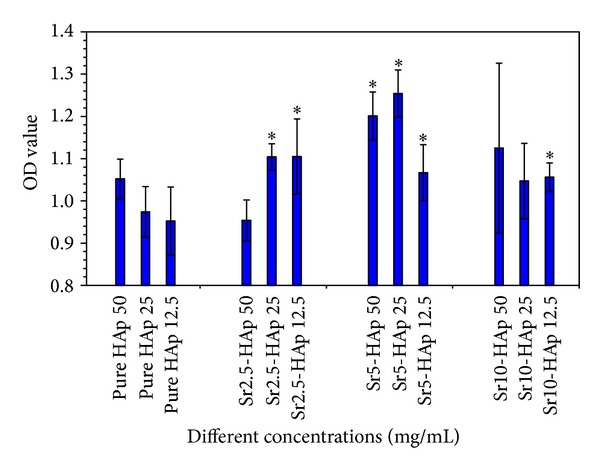
The effect of ionic products from HAp and Sr-substituted HAp whiskers on proliferation of MG-63 after 5 days of culture. ∗ indicates the experimental group compared with the control group of HAp whiskers at the same concentration, *P < 0.05.

Compared with pure HAp, a stimulatory effect of the ionic products from Sr-substituted HAp whiskers at appropriate concentrations on MG-63 proliferation was observed (Figure 4). The stimulatory effects of the ionic product from Sr5-HAp whiskers on MG-63 proliferation were apparently higher than other materials between the extract concentrations of 12.5 and 50 mg/mL. The results indicated the better therapeutic potential of Sr5-HAp whiskers for bone regeneration. Previous studies suggested that the effects of Sr on bone cells were dose-dependent [25]. The ICP-OES analysis showed that the Sr ion concentrations of the Sr5-HAp whisker extracts (12.5–50 mg/mL) for cell culture were 2.88 × 10^−3^–1.15 × 10^−2^ mmol/L. The study of Bonnelye et al. confirmed that Sr ranelate with Sr^2+^ concentrations around 0.1–1 mmol/L could promote osteoblast formation [26]. In addition, the concentration of Sr in normal serum varied between 1.14 × 10^−4^ and 2.48 × 10^−3^ mmol/L [27], which was much lower than those in our study and the reported references. Moreover, the amount of Sr in bone tissue is apparently higher than that in blood, and almost 99% of the absorbed Sr is deposited in bone (36–140 mg/kg) [25, 28]. Therefore, it can be indicated that a certain high dose of Sr may stimulate the proliferation of osteoprogenitor cells and benefit new bone regeneration [25].

## 4. Conclusions

In this study, the ultralong SrHAp whiskers with 0–5.83 mol % of Ca substituted by Sr and length up to 200 *μ*m have been successfully hydrothermally prepared using acetamide as homogeneous precipitation reagent. The Sr substitution level of the whiskers could be well facilely regulated by tailoring the initial molar ratio of Sr/(Sr + Ca) in the raw materials. In addition, the Sr^2+^ ions replaced part of Ca^2+^ ions, and the lattice constants increased apparently with the increase of the Sr substitution amount. The cell culture results showed that the ionic products of SrHAp whiskers apparently promoted the proliferation of MG-63 at certain concentrations of Sr^2+^ ions. Particularly the Sr5-HAp component was optimal for cell activity. Our study suggests that the Sr-substituted HAp whiskers might be a potential candidate as a new bioactive material in biomedical field.

## Figures and Tables

**Table 1:** Lattice constants, 2*θ* value for (3 0 0) diffraction, chemical composition, and size of the synthetic HAp and Sr-substituted HAp whiskers.

Samples	Lattice constants		2*θ* (°) for (3 0 0) reflection	Chemical composition		Size of the whiskers	
	*a* (Å)	*c* (Å)		Ca replacement by Sr (mol.%)	(Ca + Sr)/P moler ratio	Length (*μ*m)	Diameter (*μ*m)
HAp	9.423	6.915	32.900	0	1.62	33.64 ± 19.10	2.19 ± 1.84
Sr2.5HAp	9.439	6.883	32.841	1.37	1.65	83.09 ± 46.24	3.21 ± 1.58
Sr5HAp	9.445	6.912	32.821	2.72	1.63	86.36 ± 43.28	3.53 ± 1.37
Sr10HAp	9.451	6.964	32.800	5.83	1.61	95.76 ± 37.65	3.45 ± 1.26
